# The influence of the da Vinci surgical robot on electromagnetic tracking in a clinical environment

**DOI:** 10.1007/s11701-023-01812-7

**Published:** 2024-01-27

**Authors:** L. Aguilera Saiz, H. C. Groen, W. J. Heerink, T. J. M. Ruers

**Affiliations:** 1 Department of Surgical Oncology, Netherlands Cancer Institute, 1066 CX Amsterdam, The Netherlands; 2 Faculty of Science and Technology (TNW), Nanobiophysics Group (NBP), University of Twente, 7500 AE Enschede, The Netherlands

**Keywords:** Surgical navigation, Electromagnetic tracking, Robotic surgery, Position accuracy

## Abstract

Robot-assisted surgery is increasingly used in surgery for cancer. Reduced overview and loss of anatomical orientation are challenges that might be solved with image-guided surgical navigation using electromagnetic tracking (EMT). However, the robot’s presence may distort the electromagnetic field, affecting EMT accuracy. The aim of this study was to evaluate the robot’s influence on EMT accuracy. For this purpose, two different electromagnetic field generators were used inside a clinical surgical environment: a table top field generator (TTFG) and a planar field generator (PFG). The position and orientation of sensors within the electromagnetic field were measured using an accurate in-house developed 3D board. Baseline accuracy was measured without the robot, followed by stepwise introduction of potential distortion sources (robot and robotic instruments). The absolute accuracy was determined within the entire 3D board and in the clinical working volume. For the baseline setup, median errors in the entire tracking volume within the 3D board were 0.9 mm and 0.3° (TTFG), and 1.1 mm and 0.4° (PFG). Adding the robot and instruments did not affect the TTFG’s position accuracy (*p* = 0.60), while the PFG’s accuracies decreased to 1.5 mm and 0.7° (*p* < 0.001). For both field generators, when adding robot and instruments, accuracies inside the clinical working volume were higher compared to the entire tracking 3D board volume, 0.7 mm and 0.3° (TTFG), and 1.1 mm and 0.7° (PFG). Introduction of a surgical robot and robotic instruments shows limited distortion of the EMT field, allowing sufficient accuracy for surgical navigation in robotic procedures.

## Introduction

Robotic surgery has become popular due to the high quality and stable camera platform, the free-moving multijoint tools, and better ergonomics compared to laparoscopic surgery [1, 2]. However, the lack of tangible feedback and anatomical orientation are still issues to be resolved [3]. Specifically, to locate small tumor lesions, surgeons need to memorize the anatomy based on preoperative images, which can be challenging in cases of complicated anatomy. To solve this, image-guided surgical navigation (IGSN) is used, providing the surgeon with a detailed 3D anatomical road map, with real-time correlation of the operative field to the preoperative image data [4]. To navigate within this 3D road map, surgical instruments are tracked, which enables visualization of the surgical instruments in relation to the anatomical structures in the 3D road map.

In IGSN, two types of 3D tracking systems are used: optical tracking (OT) systems and electromagnetic tracking (EMT) systems. In OT, a stereo near-infrared camera is used to identify optical markers on the patient and surgical instruments. However, this requires a direct line of sight to all optical markers, which is often difficult in narrow spaces, such as during pelvic surgery [5]. In contrast, EMT does not require a direct line of sight; here, the pose of wired sensors is estimated based on an electromagnetic (EM) field. Nevertheless, distortions of the EM field, caused by, e.g., ferromagnetic materials, can locally affect the tracking accuracy [6].

The influence of minimally invasive robotic systems, like the da Vinci (DV) surgical system (Intuitive Surgical, Sunnyvale, CA), on EMT was investigated in a phantom study by Kenngott et al. [7]. Inaccuracies up to 8.5 mm were observed. A previous phantom study from our group [8] measured lower EM tracking errors of 1.8 mm in position and 1.0° in orientation. Yet, there has been no study where the position and orientation accuracy of the entire EMT volume are quantified in a standardized manner in a clinical–surgical environment.

IGSN using EMT is standard of care for complex open abdominopelvic surgeries at The Netherlands Cancer Institute (NKI-AvL) [9–12]. Preoperative images are used to create patient-specific 3D models and after intraoperative patient registration EMT allows for real-time navigation. Our next logical step is to translate the navigation procedure to robotic surgeries, by tracking the patient and robotic instruments. In this study, we evaluate the influence of the DV surgical robot on the EMT accuracy in a clinical setup, using a reproducible measuring protocol to quantify the position and orientation errors in a 3D volume. For this purpose, a custom-made 3D board that enables sampling the EM field’s tracking volume was developed. To assess the accuracy, measurements were performed on a surgical bed and the differences between three setups introducing additional distortion sources (robot and instruments) were evaluated.

## Materials and methods

### 3D measurement board

**Fig. 1:**
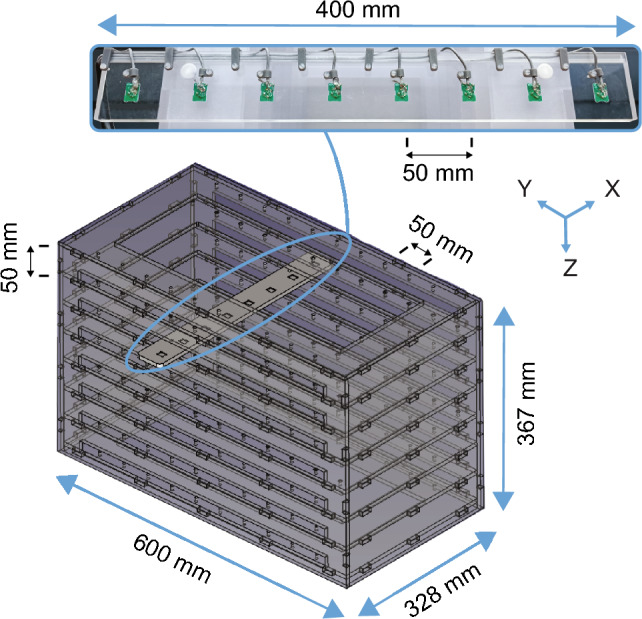
Rendering of 3D board with sensor array, constructed with 7 mm-thick polycarbonate plates. Zoomed, sensor array with eight embedded 6DOF sensors, placed on top of the 3D board

A custom-made fixed-size 3D board was built using 7 mm polycarbonate plates. The size of the 3D board (400 × 600 × 367 mm) ensures that the EM field’s tracking volume can be sampled adequately (Fig. 1) at 50.0 mm intervals in all dimensions. For this, a custom-made sensor array with eight slots precisely fitting NDI Aurora (Northern Digital Inc., Waterloo, Ontario, Canada) 6 degrees of freedom (DOF) printed circuit board (PCB) sensors (ID: 610395; 10.0 × 12.8 mm) [13] was manufactured by computer numerical control (CNC). With this array, the position and orientation of eight embedded sensors can be measured simultaneously. The array has locating pins that allow it to be accurately positioned on any level of the 3D board and measure the entire volume. It was manually repositioned to 80 locations (8 levels × 10 positions), subsequently measuring 800 predefined positions and orientations.

The 3D board was manufactured within an average deviation of 0.01 mm for the height and 0.03 mm for distances between the pins. The diagonal of the 3D board’s plates was measured to confirm squareness. The 3D board construction accuracy was validated on a surface plate using a height gauge (Vernier Height Gauge 506-205, Mitutoyo) for the height and a caliper (160-127 Nib Jaw Fine Adjustment Vernier Caliper 0–300 mm, Mitutoyo) for the distance between the locating pins.

### Experiment design

**Fig. 2:**
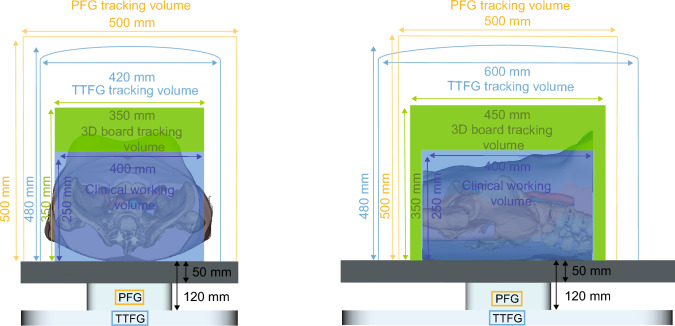
Definition of tracking volume of the 3D board (*green*) and the clinical working volume (*dark blue*) in the left–right direction (*left*) and cranial–caudal direction (*right*) with overlaid EMT volumes (TTFG, *light blue* and PFG, *yellow*) (colour figure online)

The accuracy of the NDI Aurora EMT system was measured using two different field generators (FGs), i.e., table top field generator (TTFG, V3) and the planar field generator (PFG, V2) (Fig. 2). The TTFG has an oval EMT measurement volume of 420 × 600 × 480 mm (X, Y, Z dimensions) and tracking starts from 120 mm above the FG, with a reported accuracy of 0.8 mm and 0.7° at the center of the FG. The PFG has a cubic EMT measurement volume of 500 × 500 × 500 mm (X, Y, Z dimensions) starting from 50 mm above the FG, with a reported accuracy of 0.5 mm and 0.3° at the center of the FG. The tracking volume the 3D board can measure is 350 × 450 × 350 mm (X, Y, Z dimensions). To focus toward clinical relevance in the abdominopelvic region, a 350 × 400 × 250 mm (X, Y, Z dimensions) clinical volume within the 3D board’s tracking volume was defined.

Data was acquired using NDI Track (NDI ToolBox 5.002.022) software, recording eight (X direction) 6-DOF sensors simultaneously with the default acquisition rate of 40 Hz. At each location, 250 measurements were acquired for each of the sensors. The sensor board was positioned at ten positions (Y direction) at eight levels (Z direction), resulting in a grid of 8 × 10 × 8 measurements—total 640 locations—spaced 50 mm from each other.

**Fig. 3:**
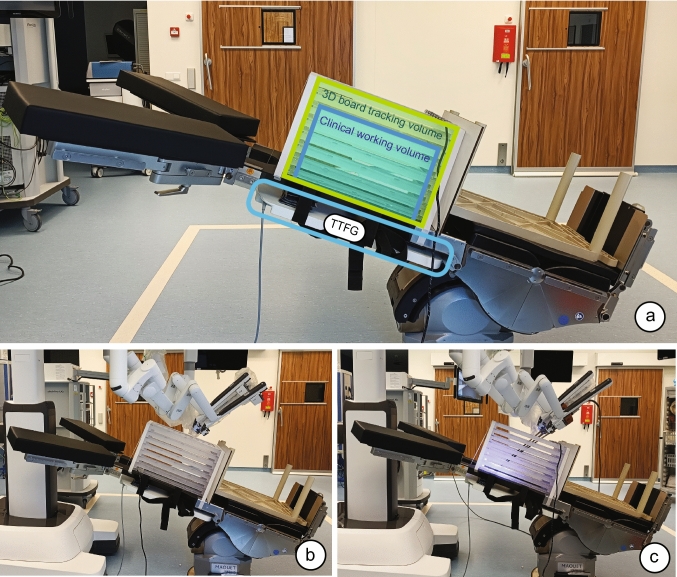
Measurement setups using the TTFG placed underneath the carbon fiber insert: baseline (**a**), robot positioned (**b**) and robot with instruments (**c**). The 3D board is positioned on a surgical table in Trendelenburg

The measurements were conducted simulating a clinical setup for abdominopelvic robotic surgeries inside the operating theater. In these kinds of procedures, the surgical table is positioned in Trendelenburg. The inclination of the surgical table (Trendelenburg position) helps the organs move cranially and facilitates more space for operating in the pelvic cavity. Positioning the DV Xi surgical system between the leg supports of the surgical table allows ergonomic access to the pelvic anatomy. To minimize ferromagnetic distortions, i.e., the surgical table and column, the surgical table is adapted with a carbon fiber multipurpose plate (Fig. 3) (118044AC, Getinge AB, Göteborg, Sweden). The FGs are positioned flat underneath the multipurpose carbon fiber plate, and the 3D board is placed directly on top of the multipurpose plate. In total, three different measurement setups were used: baseline (surgical table only), robot positioned between the legs, and robot between the legs with instruments. The instruments used—placed corresponding to the configuration of a robotic prostatectomy—were the Prograsp Forceps in arm 1, the Maryland Bipolar Forceps in arm 2, the endoscope in arm 3 and the large needle driver in arm 4.

### Data analysis

Data were evaluated with Matlab R2021a using custom-made scripts. In the analysis, measurements outside the EM tracking volume were removed from the dataset. After initial alignment, measurements were registered to the 3D board using an iterative closes point (ICP) algorithm [14].

Accuracy was calculated following the ISO 5725-1 [15] standard, where it is defined as a combination of trueness and precision. Position errors were calculated using the root mean square error (RMSE) of the Euclidian distance between the averaged measured position and the registered 3D board position. The orientation RMSE in angles was calculated by the quaternion difference between the measured orientation and the truth orientation. The latter is defined to be the orientation in the most accurate row of sensors in the EM tracking volume, i.e., (*Y* = 0, *Z* = 270) for the TTFG and (*Y* = 0, *Z* = 90) for the PFG. The precision (jitter) for both position and orientation was calculated by the RMSE of the difference between all 250 measurements per location and the mean over all measurements at that location.

Statistical analysis was performed using Matlab R2021a. A Shapiro–Wilk test was used to determine if data were normally distributed. A Kruskal–Wallis one-way ANOVA test with Bonferroni correction was used to assess the difference between the three setups. Here, a *p* < 0.01 was considered a statistically significant difference. Data are presented as RMSE median (Q1–Q3) and visualized using boxplots with median, IQR (Q1–Q3), min–max and outliers as well as a point cloud.

## Results

### Entire 3D board measurement volume

There were no significant differences between the setups for both the position jitter (all <0.2 mm) and the orientation jitter (all <0.1°) (data not shown). Median position errors of the TTFG were 0.9 mm for all the setups, for the PFG median position error increased from 1.1 mm at baseline to 1.5 mm in the robot with instruments setup. Orientation errors of the TTFG increased only slightly from 0.3° at baseline to 0.4° in the robot and instruments setup. For the PFG, orientation errors increased from 0.4° at baseline to 0.7° in the robot and instruments setup.

**Fig. 4:**
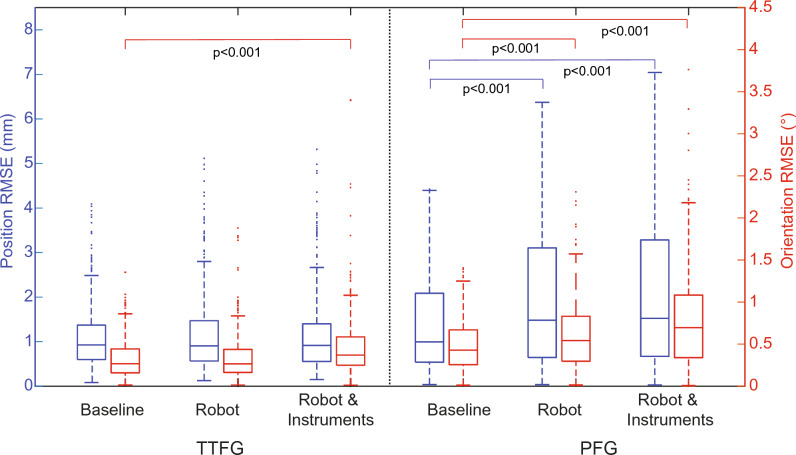
Boxplots of the position (*blue*) and orientation (*red*) RMSE per experiment, calculated from the 250 measurements mean from the entire 3D board tracking volume (colour figure online)

Data were not normally distributed for all measurements (Fig. 4). On average, for the TTFG, there was no significant difference of the position error between the baseline and the robot setup (*p* = 0.98), or with the addition of the instruments (*p* = 0.60). Although there was also no significant difference in the orientation between baseline and robot setups (*p* = 0.78), there was a slight difference between the baseline and robot with instruments setup (*p* < 0.001). For the PFG, the position and orientation error increased significantly when adding the robot (*p* < 0.001 for position and orientation) and the robot with instruments (*p* < 0.001 for position and orientation).

**Fig. 5:**
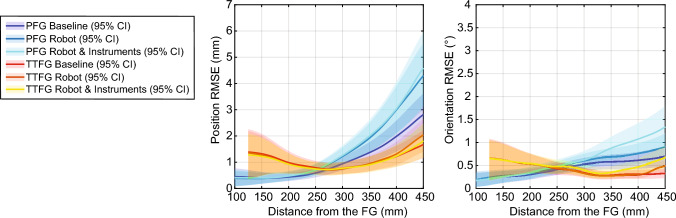
Position (*left*) and orientation (*right*) RMSE as function of the distance to the FG (0, 0, 0), with the 95% confidence intervals. Data from the TTFG is displayed in warm colors (*red*, *orange*, and *yellow*), and the PFG data in cold colors (*purple*, *dark blue*, and *light blue*) (colour figure online)

**Fig. 6:**
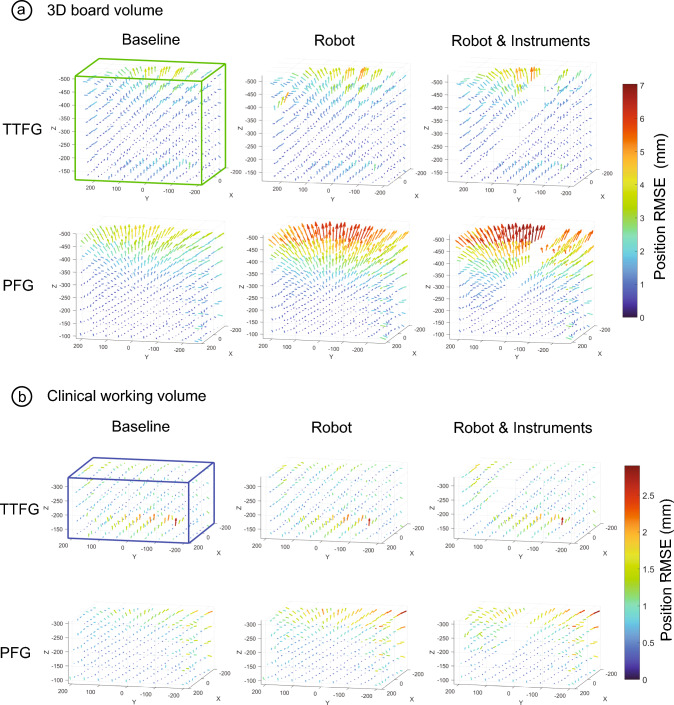
Position RMSE at **a** the entire 3D tracking volume (green bounding box) and **b** the clinically working volume (*blue bounding box*) from the registered point cloud of the TTFG (*top*) and PFG (*bottom*): baseline (*left*), robot (*middle*) and robot with instruments (*right*). The robot is positioned in the positive direction of the Y axis (*left* from the *graphs*). The *arrows* show the error direction scaled times 10 for visualization and the size the value of the error (*color coded*) in mm (colour figure online)

Both position and orientation errors are dependent on the distance from the FG, as visualized in Figs. 5 and 6a. Especially for the PFG, accuracy decreased at a further distance from the FG.

### Clinical working volume

Figure 6b shows a cropped view of the position RMSE at the clinical working volume. For the TTFG, median (Q1–Q3) position error was 0.7 (0.5–1.0) mm for all the setups. For the PFG, position error was 0.5 (0.4–0.8) mm for the baseline setup, 0.7 (0.4–1.1) mm for the robot setup, and 0.8 (0.4–1.2) mm for the robot with instruments setup.

In the clinical working volume, orientation errors of the TTFG were below 0.3 (0.1–0.5)° for all the setups, and below 0.7 (0.3–1.2)° for the PFG.

## Discussion

This study’s primary objective was to determine the influence of the DV surgical robot on the accuracy of the NDI Aurora EMT system. Among several existing accuracy measurement protocols [16], the most common standardized protocol to evaluate EMT accuracy is the Hummel board [17] with the Hummel protocol [18]. However, the Hummel board lacks reproducible measurements at different heights. This has been solved in our measurement board, which enables measuring the entire tracking field in 3D. This allows for highly reproducible and accurate position measurements of EMT FGs in different clinical setups.

The DV Xi surgical robot was tested with two different NDI Aurora FGs (PFG and TTFG) in a clinical setup. When using the TTFG, adding the robot and instruments led to no significant differences in position accuracy and a minimal increase in orientation accuracy of 0.1° (*p* < 0.001) when introducing instruments. For the PFG, errors increased by 0.4 mm and 0.3° (*p* < 0.001). Jitter of both position and orientation was low, i.e., <0.2 mm and <0.1°, and showed no significant difference (*p* < 0.001) between setups. When using the TTFG, median RMSE for position and orientation was 0.9 (0.6–1.5) mm and 0.4 (0.2–0.6)° when using the robot in full extent with instruments. For the PFG, these values were 1.5 (0.7–3.3) mm and 0.7 (0.3–1.1)°, respectively. The highest errors were present in the upper part of the EM volume, further from the FG. However, this is outside the volume that is generally clinically relevant to track for robotic surgery. Within the defined clinical working volume, median accuracies for the position were equal or below 0.8 (0.4–1.2) mm and below 0.7 (0.3–1.2)° for orientation. Here, the higher limit of error was close to the field generator for the TTFG, and at the outer border of the defined clinical working volume for the PFG. This can probably be explained by the FG’s design. Manufacturers’ accuracies are 0.8 mm and 0.7° for the TTFG and 0.5 mm and 0.3° for the PFG. Both errors measured in the current study are thereby acceptable for clinical use.

In a previous study, Kenngott et al. [7] used an NDI Aurora EMT system to assess the distortion caused by a DV surgical robot in a clinical environment. They concluded that the surgical table caused the greatest interference with their setup resulting in a position inaccuracy of 8.5 mm and did not analyze the orientation. Due to the different study setup, it is difficult to make a direct comparison with Kenngott et al. and our results. Yet, adapting our surgical table—non-ferromagnetic insert with the FG underneath—we measured a 1.5 mm position inaccuracy. Another advantage of this insert is that the FG is further away from the table’s column, containing a high amount of ferromagnetic materials, which might have contributed to the lower accuracy.

Although robot motor activation can be expected to affect the EM field, the dynamic measurements from Kenngott et al. [7] demonstrated that the robot arm movements did not influence tracking accuracy. Additionally, in our experience, navigation with EMT is used in a quasi-static way; the surgeon only looks at the navigation interface when most instruments are in a steady position and at most one (tracked) instrument makes small movements. Nevertheless, the static robot arm setup of the current study is a limitation, when considering using EMT with robotic surgery in a more dynamic setting.

Optical tracking with near-infrared cameras with submilimetric accuracy has been used in patient case studies for robotic IGSN [19]. However, the optical camera requires constant line of sight with the optical trackers for this technique. As indicated by the authors, the sterile drapes and instruments can easily obstruct the camera’s view. Furthermore, instrument tracking is more accurate when the trackers are close to the tip, which is not possible using optical trackers inside the body. Therefore, the proposed EMT is more suitable for navigation in robotic surgery.

In our study, the orientation analysis was conducted at a single angle because of our current 3D board’s configuration, with all sensors positioned in a planar arrangement parallel to FG. This could lead to unforeseen orientation errors in other arrangements. In addition, we used 6 DOF NDI Aurora sensors, which are the most accurate ones currently available. These sensors are suitable to accurately quantify the EM field, but for other (smaller) sensors a slightly higher error can be expected. Furthermore, the effect of cautery devices on tracking accuracy was not investigated. In our experience, the use of cautery equipment generally causes extreme jitter, preventing the use of navigation. However, the moment cauterization is stopped, tracking accuracy returns to normal allowing continuation of navigation.

## Conclusion

To our knowledge, this is the first study that quantifies the influence of a surgical robot on the accuracy of an EMT system in the 3D tracking volume applied for surgical navigation. We have shown that using NDI Aurora with a DV Xi surgical robot is accurate in clinical environments optimized for EMT, with an accuracy in the clinical working volume below 1.0 mm in position and 1.0° in orientation. Integrating EMT with new generations of DV surgical robots is potentially feasible.

## Data Availability

The data are not publicly available. The data that support the findings of this study are available from the corresponding author, LAS, upon reasonable request.

## References

[1] Kim MJ, Park SC, Park JW, Chang HJ, Kim DY, Nam BH, Sohn DK, Oh JH (2018) Robot-assisted versus laparoscopic surgery for rectal cancer: a phase II open label prospective randomized controlled trial. Ann Surg 267:243–251. 10.1097/SLA.0000000000002321 28549014 10.1097/SLA.0000000000002321

[2] Li X, Wang T, Yao L, Hu L, Jin P, Guo T, Yang K (2017) The safety and effectiveness of robot-assisted versus laparoscopic TME in patients with rectal cancer. Medicine (United States) 96. 10.1097/MD.0000000000007585 10.1097/MD.0000000000007585 PMC5521938 28723798

[3] Wottawa CR, Genovese B, Nowroozi BN, Hart SD, Bisley JW, Grundfest WS, Dutson EP (2016) Evaluating tactile feedback in robotic surgery for potential clinical application using an animal model. Surg Endosc 30:3198–3209. 10.1007/s00464-015-4602-2 26514132 10.1007/s00464-015-4602-2 PMC4851934

[4] Cheng S, Li X, Zhu W, Li W, Wang J, Yang J, Wu J, Wang H, Zhang L, Li X, Zhou L (2021) Real-time navigation by three-dimensional virtual reconstruction models in robot-assisted laparoscopic pyeloplasty for ureteropelvic junction obstruction: our initial experience. Transl Androl Urol 10:125–133. 10.21037/TAU-20-1006 10.21037/tau-20-1006 PMC7844525 33532302

[5] Nijkamp J, Schermers B, Schmitz S, de Jonge S, Kuhlmann K, van der Heijden F, Sonke JJ, Ruers T (2016) Comparing position and orientation accuracy of different electromagnetic sensors for tracking during interventions. Int J Comput Assist Radiol Surg 11:1487–1498. 10.1007/s11548-015-1348-1 26811081 10.1007/s11548-015-1348-1

[6] Frantz DD, Wiles AD, Leis SE, Kirsch SR (2003) Accuracy assessment protocols for electromagnetic tracking systems. Phys Med Biol 48:2241–2251. 10.1088/0031-9155/48/14/314 12894982 10.1088/0031-9155/48/14/314

[7] Kenngott HG, Wegner I, Neuhaus J, Nickel F, Fischer L, Gehrig T, Meinzer HP, Müller-Stich BP (2013) Magnetic tracking in the operation room using the da Vinci® telemanipulator is feasible. J Robot Surg 7:59–64. 10.1007/s11701-012-0347-2 23440620 10.1007/s11701-012-0347-2 PMC3574972

[8] Aguilera Saiz L, ten Bolscher W, Groen HC, Heerink WJ, Hiep M, Ruers TJM (2022) Towards surgical navigation with electromagnetic tracking for robotic surgeries. Int J Comput Assist Radiol Surg 1–147. 10.1007/s11548-022-02635-x

[9] Nijkamp J, Kuhlmann KFD, Ivashchenko O, Pouw B, Hoetjes N, Lindenberg MA, Aalbers AGJ, Beets GL, van Coevorden F, KoK N, Ruers TJM (2019) Prospective study on image-guided navigation surgery for pelvic malignancies. J Surg Oncol 119:510–517 30582622 10.1002/jso.25351

[10] Nijkamp J, Kuhlmann K, Sonke J-J, Ruers T (2016) Image-guided navigation surgery for pelvic malignancies using electromagnetic tracking. Med Imaging 2016 Image-Guided Proced Robot Interv Model 9786:97862L. 10.1117/12.2216213

[11] Kok END, Van Veen R, Groen HC, Heerink WJ, Hoetjes NJ, Van Werkhoven E, Beets GL, Aalbers AGJ, Kuhlmann KFD, Nijkamp J, Ruers TJM (2020) Association of image-guided navigation with complete resection rate in patients with locally advanced primary and recurrent rectal cancer: a nonrandomized controlled trial. JAMA Netw Open 3:1–11. 10.1001/jamanetworkopen.2020.8522 10.1001/jamanetworkopen.2020.8522 PMC7344384 32639566

[12] Groen HC, Den Hartog AG, Heerink WJ, Kuhlmann KFD, Kok NFM, van Veen R, Hiep MAJ, Snaebjornsson P, Grotenhuis BA, Beets GL, Aalbers AGJ, Ruers TJM (2022) Use of image-guided surgical navigation during resection of locally recurrent rectal cancer. Life 12:1–13. 10.3390/life12050645 10.3390/life12050645 PMC9143650 35629313

[13] 610395 DS (2019) Aurora 6DOF sensor PCB assembly 12.8 × 10 mm:10–13. https://support.ndigital.com/s/download-page. Accessed 16 Aug 2023

[14] Wilm J (2023) Iterative closest point. In: MATLAB Cent. File Exch. https://www.mathworks.com/matlabcentral/fileexchange/27804-iterative-closest-point. Accessed 16 Aug 2023

[15] International Organization for Standarization I (1994) Accuracy (trueness and precision) of measurement methods and results Iso 5725–1, 1st ed. Geneva

[16] Franz AM, Haidegger T, Birkfellner W, Cleary K, Peters TM, Maier-Hein L (2014) Electromagnetic tracking in medicine—a review of technology, validation, and applications. IEEE Trans Med Imaging 33:1702–1725. 10.1109/TMI.2014.2321777 24816547 10.1109/TMI.2014.2321777

[17] Sirokai B, Kiss M, Kovács L, Benyó B, Benyó Z, Haidegger T (2012) Best Practices in electromagnetic tracking system assessment. Proc Jt Work New Technol Comput Assist Surg (SCATh), Madrid ID:12:1–4

[18] Haidegger T, Sirokai B, Fenyvesi G, Kovács L, Benyó B, Benyó Z (2012) Repeatable assessment protocol for electromagnetic trackers. Med Imaging 2012 Image-Guided Proced Robot Interv Model 8316:83161S. 10.1117/12.911673

[19] Atallah S, Parra-Davila E, Melani AGF, Romagnolo LG, Larach SW, Marescaux J (2019) Robotic-assisted stereotactic real-time navigation: initial clinical experience and feasibility for rectal cancer surgery. Tech Coloproctol 23:53–63. 10.1007/s10151-018-1914-y 30656579 10.1007/s10151-018-1914-y

